# Identification and validation of autophagy-related gene expression for predicting prognosis in patients with idiopathic pulmonary fibrosis

**DOI:** 10.3389/fimmu.2022.997138

**Published:** 2022-09-20

**Authors:** Guichuan Huang, Xin Xu, Chunrong Ju, Nanshan Zhong, Jianxing He, Xiao Xiao Tang

**Affiliations:** ^1^ State Key Laboratory of Respiratory Disease, National Clinical Research Center for Respiratory Disease, National Center for Respiratory Medicine, Guangzhou Institute of Respiratory Health, The First Affiliated Hospital of Guangzhou Medical University, Guangzhou, China; ^2^ Guangzhou Laboratory, Guangzhou, China

**Keywords:** idiopathic pulmonary fibrosis, prognostic model, autophagy, MET, SH3BP4

## Abstract

**Background:**

Idiopathic pulmonary fibrosis (IPF) is a chronic, progressive, and fatal fibrotic pulmonary disease with unknow etiology. Owing to lack of reliable prognostic biomarkers and effective treatment measures, patients with IPF usually exhibit poor prognosis. The aim of this study is to establish a risk score prognostic model for predicting the prognosis of patients with IPF based on autophagy-related genes.

**Methods:**

The GSE70866 dataset was obtained from the gene expression omnibus (GEO) database. The autophagy-related genes were collected from the Molecular Signatures Database (MSigDB). Gene enrichment analysis for differentially expressed genes (DEGs) was performed to explore the function of DEGs. Univariate, least absolute shrinkage and selection operator (LASSO), as well as multivariate Cox regression analyses were conducted to identify a multi-gene prognostic model. Receiver operating characteristic (ROC) curve was applied to assess the prediction accuracy of the model. The expression of genes screened from the prognostic model was validated in clinical samples and human lung fibroblasts by qPCR and western blot assays.

**Results:**

Among the 514 autophagy-related genes, a total of 165 genes were identified as DEGs. These DEGs were enriched in autophagy-related processes and pathways. Based on the univariate, LASSO, and multivariate Cox regression analyses, two genes (MET and SH3BP4) were included for establishing the risk score prognostic model. According to the median value of the risk score, patients with IPF were stratified into high-risk and low-risk groups. Patients in high-risk group had shorter overall survival (OS) than low-risk group in both training and test cohorts. Multivariate regression analysis indicated that prognostic model can act as an independent prognostic indicator for IPF. ROC curve analysis confirmed the reliable predictive value of prognostic model. In the validation experiments, upregulated MET expression and downregulated SH3BP4 expression were observed in IPF lung tissues and TGF-β1-activated human lung fibroblasts, which is consistent with results from microarray data analysis.

**Conclusion:**

These findings indicated that the risk score prognostic model based on two autophagy-related genes can effectively predict the prognosis of patients with IPF.

## Introduction

Idiopathic pulmonary fibrosis (IPF) is a chronic, progressive and fatal interstitial lung disease with unknown etiology (1). It is characterized by repetitive epithelial cell injury, fibroblast activation and overwhelming extracellular matrix (ECM) deposition which ultimately cause progressive loss of lung function and even death owing to respiratory failure (1). In the USA, the annual occurrence rate of IPF was 6.8-8.8 per 100,000 population with narrow case definitions and 16.3-16.7 per 100,000 population with broad case definitions (2). The annual occurrence rate in Europe was 0.22-7.4 per 100,000 population (2). The medial survival after diagnosis is only 2-3 years and the 5-year survival rate is no more than 40% (3, 4). Due to the complex etiology and unclear pathogenesis, there is still a lack of effective drugs. Pirfenidone and nintedanib, the two FDA-approved drugs, can’t stop disease progression or reduce mortality (5). Therefore, it is important to identify the pathogenesis of IPF, explore novel treatment strategies and develop prognosis model.

Autophagy is a multi-step dynamic process that regulated by autophagy-related genes. In this process, autophagosomes are generated by phagocytosis of unwanted organelles and cytoplasmic proteins in a double membraned-surrounded vesicle (6). Then, autophagosomes are fused with lysosomes to degrade the contents of vesicles (6). Dysregulation of autophagy is involved in various lung diseases, including pulmonary hypertension, asthma, chronic obstructive pulmonary disease, and pulmonary fibrosis (7). For instance, a study has shown that leucine-rich repeat kinase 2 (LRRK2) is conducive to alleviate pulmonary fibrosis *via* preventing alveolar type II epithelial dysfunction and regulating the innate immune responses (8). Kim et al. reported that interleukin-37 (IL-37) attenuates IPF by blocking the transforming growth factor-β1 (TGF-β1) pathway and enhancing autophagy in IPF fibroblast (9). Wan et al. found that the downregulation of thymocyte differentiation antigen-1 (Thy-1) and upregulation of integrin β3 can lead to pulmonary fibrosis *via* activating PI3K/AKT/mTOR pathway and inhibiting lung fibroblast autophagy (10). Nevertheless, the role of autophagy-related genes in the prognosis of IPF remains largely unclarified and awaits further study.

In the present study, the autophagy-associated differentially expressed genes (DEGs) between control samples and IPF samples were analyzed in GSE70866 dataset. Gene ontology (GO) and kyoto encyclopedia of genes and genomes (KEGG) enrichment analyses were performed for DEGs. Then, based on the univariate, least absolute shrinkage and selection operator (LASSO) as well as multivariate regression analyses, two autophagy genes were included to establish a risk score prognostic model in the training set. Finally, this risk score model was proved to be an independent and reliable prognostic factor in patients with IPF.

## Materials and methods

### Acquisition of dataset and autophagy-related genes

The microarray data and clinical information in GSE70866 dataset (GPL14550 platform) were downloaded from gene expression omnibus (GEO) database [20 normal bronchoalveolar lavage fluid (BALF) samples and 112 IPF BALF samples]. The diagnosis of IPF in the dataset was confirmed by a multidisciplinary board at each institution according to the American Thoracic Society/European Respiratory Society criteria. To obtain BALF, pre-warmed and sterile saline was instilled by 20ml aliquots with immediate aspiration by gentle suction after each aliquot. Additional information regarding the collected BALF samples can be seen in this article (11). The raw microarray data were pre-processed for quality control with the use of “limma” package, including background adjustment and normalization. The general information of 112 patients with IPF was presented in **Table 1**. A total of 12 autophagy-related gene sets were downloaded from the Molecular Signatures Database (MSigDB) (version 7.5.1) (**Supplementary Table 1**). After deleting the overlapping genes, 504 autophagy-associated genes were included for analysis (**Supplementary Table 2**).

**Table 1:** Baseline characteristic of patients with IPF in training and test set.

Characteristic	Total (n=112)	Training set (n=56)	Test set (n=56)	*P* value
**Age (Mean±SD)**	67.97±10.06	69.54±10.36	66.41±9.61	0.812
**Age, n (%)**				
<70	56 (50.00)	25 (44.64)	31 (5.36)	
≥70	56 (50.00)	31 (55.36)	25 (44.64)	0.257
**Sex, n (%)**				
Male	93 (83.04)	44 (78.57)	49 (87.50)	
Female	19 (16.96)	12 (21.43)	7 (12.50)	0.208
**GAP index (Mean±SD)**	4.54±1.73	4.64±1.88	4.43±1.58	0.535
**Status, n (%)**				
Alive	36 (32.14)	19 (33.93)	17 (30.36)	
Dead	76 (67.86)	37 (66.07)	39 (69.64)	0.686

SD, Standard deviation; GAP, Gender-age-physiology.

### Identification of autophagy-associated DEGs

The autophagy-associated DEGs between normal samples and IPF samples were investigated using “limma” R package. A gene with *p*<0.05 was considered as DEG. Then, based on the GO and KEGG analyses, the biological functions and mechanism pathways for these autophagy-associated DEGs were explored.

### Construction of risk score prognostic model

A total of 112 IPF samples was randomly divided into the training set (n=56) and the test set (n=56) with the use of “caret” R package. First, the prognosis-related genes from autophagy-associated DEGs were identified utilizing a univariate Cox regression analysis in the training set. Then, in order to avoid overfitting, we adopted the LASSO regression analysis to obtain the crucial autophagy-associated DEGs. Finally, a multivariate Cox regression analysis was conducted to select the autophagy-associated genes for establishing a risk score prognostic model. The formula of risk score model was presented as follows: Risk score= [(expression value of gene 1 × β1) + (expression value of gene 2 × β2) +…+ (expression value of gene n × βn)], where β is the corresponding gene’s regression coefficient. The risk score of each sample was calculated according to the formula. Samples were stratified into the high-risk group and low-risk group on the basis of the median value of risk score. Kaplan-Meier analysis and log-rank test were performed to compare the survival differences between the two risk groups using “survival” R package. Receiver operating characteristic (ROC) curve was conducted to assess the model’s prediction accuracy using “survivalROC” R package. Cox regression analysis, including univariate and multivariate, was performed to evaluate whether the risk score model is an independent factor in IPF.

### Pre and post risk score prognostic model comparison for principal component analysis

First, based on all autophagy-associated genes, PCA was performed to explore the sample distribution between two risk groups in the training set. Then, based on the two genes from risk score model, PCA was performed again. Finally, the “ggplot2” R package was employed to visualize the results.

### The relationship between risk scores and clinical parameters

The relationship between risk scores and clinical parameters was explored, including age, gender, gender-age-physiology (GAP) index. GAP index is a staging system for patients with IPF and can be calculated using gender (G), age (A), and two lung physiology variables (P), including forced vital capacity (FVC) and diffusing capacity for carbon monoxide (DLCO) (12). GAP index may be used as a simple and quick approach for assessing risk in patients with IPF (12). IPF samples were divided into different groups according to the clinical parameters and the difference of risk scores among these groups was compared.

### Immune cell infiltration and immune-related function analyses in the two risk groups

The 22 kinds of immune cells in two risk groups were determined using CIBERSORT. CIBERSORT is a deconvolution method that assesses the immune cell composition of tissue from their gene expression profile (13). It applies linear support vector regression (SVR) (a machine learning approach) to deconvolute a mixture of gene expression. It has been shown that the results are correlated well with flow cytometric analysis. Therefore, CIBERSORT is also referred as “digital cytometry” (14). In addition, the 13 kinds of immune-related function were explored in the two risk groups.

### Gene set variation analysis

GSVA is a nonparametric and unsupervised analysis method to condense information from gene expression profiles into a pathway summary (15). To investigate the biological process between the two risk groups, GSVA was performed with the use of “GSVA” R package. *P*<0.05 was considered as statistically significant. The gene set of “c2.cp.kegg.v7.5.1.symbols”, downloaded from the MSigDB, was used as a reference.

### Validation of the risk score model in the test set

According to the median value of risk score from the training set, the patients with IPF in test set were split into the high-risk and low-risk groups. The OS in two groups were compared using Kaplan-Meier analysis and log-rank test. ROC curve was conducted to evaluate the prediction accuracy of the model in the test set.

### Preliminary experimental validation of the genes from risk score model

Lung tissue samples were obtained from six patients with IPF and six healthy controls at the First Affiliated Hospital of Guangzhou Medical University. This study was approved by the ethics committee of the First Affiliated Hospital of Guangzhou Medical University and was carried out in accordance with the Declaration of Helsinki.

Human lung fibroblasts were purchased from the ATCC. Cells were cultured in DMEM medium supplemented with 10% fetal bovine serum, 100U/ml penicillin, and 100mg/ml streptomycin (Gibco) at 37°C in a 5% carbon dioxide atmosphere. The fibroblasts were stimulated with 10ng/ml TGF-β1 for 48h to induce them differentiate into myofibroblasts. Then, the total RNA and protein were collected for further analysis.

### qPCR

To detect the mRNA levels, total RNA from cells was obtained using NucleoZOL reagent (Macherey-nagel Gmbh & Co. Kg, Germany). RNA concentration was determined with a NanoDrop 2000 micro−spectrophotometer (Thermo Fisher Scientific, USA). Then, total RNA was reverse-transcribed into complementary DNA (cDNA) using Hifair^®^ III 1st Strand cDNA Synthesis SuperMix (Yeasen Biotechnology, China). Subsequently, the cDNA was amplified by SYBR Green Master Mix (Yeasen Biotechnology, China). The relative expression levels of mRNA were normalized to the levels of GAPDH and calculated by the 2^-ΔΔ^CT method.

The primers sequences were as follows:

MET, forward, 5’-AGCGTCAACAGAGGGACCT-3’, reverse, 5’-GCAGTGAACCTCCGACTGTATG-3’; SH3BP4, forward, 5’-ACCCTGATTGACCTGAGCGA-3’, reverse, 5’- GGGGTTGTCTACGAGCAAGG-3’.

### Western blot

Tissues from explanted IPF lungs or healthy donor lungs were collected and stored in liquid nitrogen before use. For protein extraction, tissues or cells were homogenized in ice-cold RIPA lysis buffer supplemented with phenylmethylsulfonyl fluoride (Biosharp, China) and phosphatase inhibitor cocktail (Sigma, USA). After centrifuging at 14,000 xg for 30 min at 4 °C, the supernatant was collected as total protein and the protein concentration was determined using BCA protein assay kit (Thermo Fisher Scientific, USA). Equal amounts of protein (20µg) were separated by 10% SDS−PAGE and transferred to PVDF membranes. After blocking with 5% non-fat milk at room temperature for 1h, the membranes were soaked in primary antibodies solutions at 4°C overnight. On the next day, membranes were washed with TBST for three times, then incubated with secondary antibodies at room temperature for 1h. Finally, the protein bands were visualized *via* an electrochemiluminescence reagent (Thermo Fisher Scientific, USA). The images were analyzed by Image J software. The following primary antibodies were utilized: anti-MET (1:1000, 25869-1-AP, Proteintech), anti-SH3BP4 (1:200, sc-393730, Santa Cruz).

### Statistical analysis

The statistical analysis was implemented by R software (version 4.1.3). Gene expression in two groups was compared by Wilcoxon test. Univariate Cox, LASSO and multivariate Cox regression analyses were performed to identify the prognosis-associated genes. The Kaplan-Meier analysis combined with a log-rank test was used to explore the differences in OS between two groups. ROC analysis was conducted to assess accuracy of the risk score prognostic model.

Raw data from qPCR and western blot analysis were presented as “mean ± standard error of mean (SEM)” and were further compared by Student’s t test. GraphPad software (version 8.0) was used to visualize the statistical results. *p*<0.05 was considered as statistically significant.

## Results

### Identification of autophagy-related DEGs and function enrichment analysis in IPF

**Figure 1:**
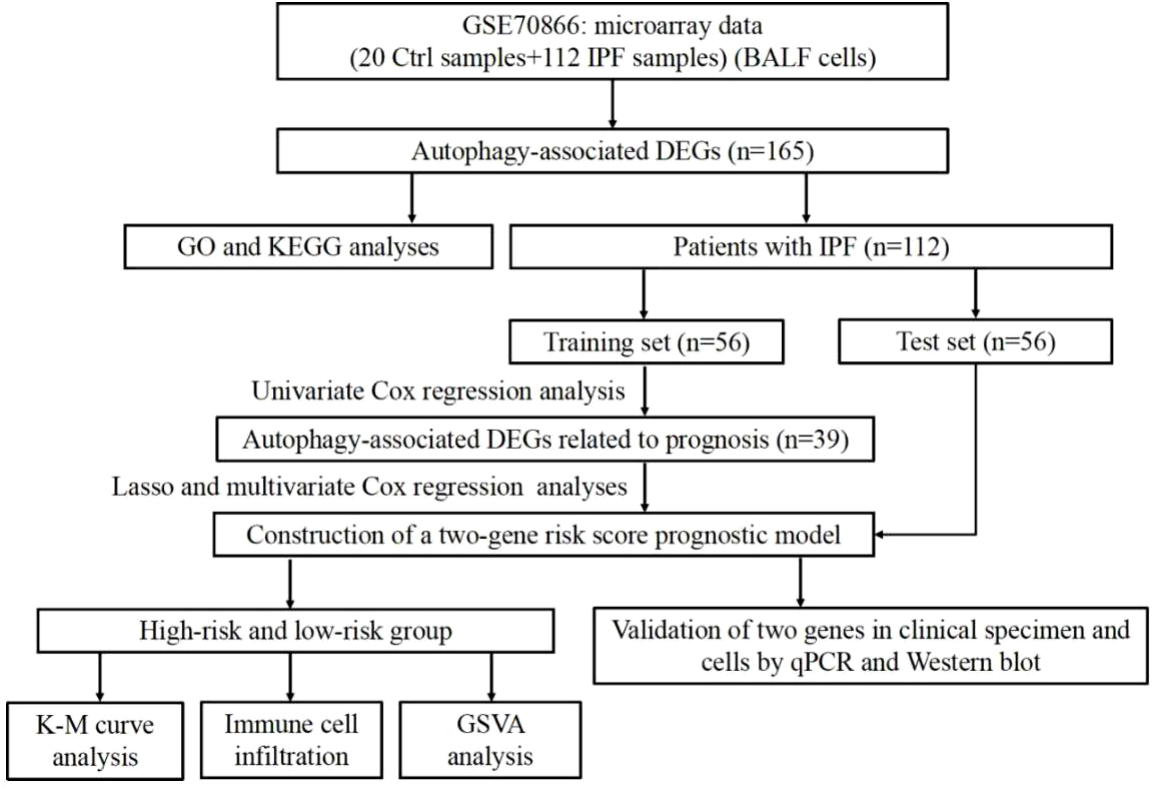
Flow chart of the study.

The overall flow chart for this study was presented in **Figure 1**. A total of 504 autophagy-related genes were collected from the MSigDB database (**Supplementary Table 2**). We explored the expression of autophagy-related genes between the 20 normal samples and 112 IPF samples. Among the 504 autophagy-related genes, 165 genes with *p*<0.05 were considered as DEGs (**Supplementary Table 3**). Of the 165 DEGs, 113 genes were downregulated and 52 genes were upregulated in IPF.

**Figure 2:**
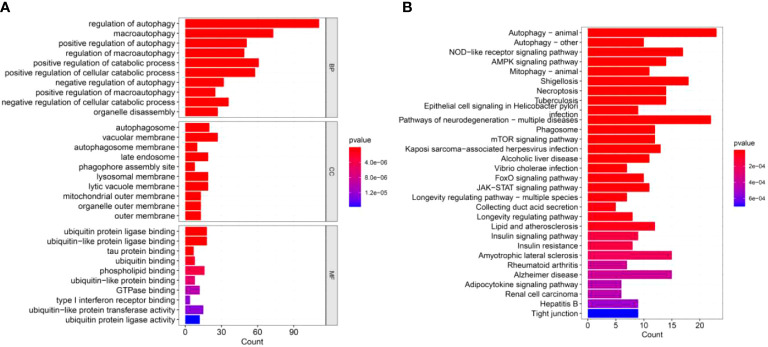
GO and KEGG enrichment analyses of DEGs. **(A)** GO enrichment analysis of DEGs, including BP, CC, and MF. **(B)** KEGG enrichment analysis of DEGs.

To further understand the function of these DEGs in IPF, we conducted GO and KEGG enrichment analyses. Three categories, including biological process (BP), cellular component (CC) and molecular functions (MF), were presented to describe the GO analysis. With respect to BP, DEGs were mainly enriched in autophagy-related biological activities (**Figure 2A**). Regarding CC, the top three items were autophagosome, vacuolar membrane, and autophagosome membrane, which were associated with autophagy (**Figure 2A**). In terms of MF, DEGs were closely related to ubiquitin protein ligase which can degrade the proteins (**Figure 2A**). For the KEGG pathways, DEGs were also enriched in autophagy-related pathways, such as mTOR, JAK-STAT, and autophagy signaling pathways (**Figure 2B**).

### Establishment of an autophagy-related gene risk score model in the training set

**Figure 3:**
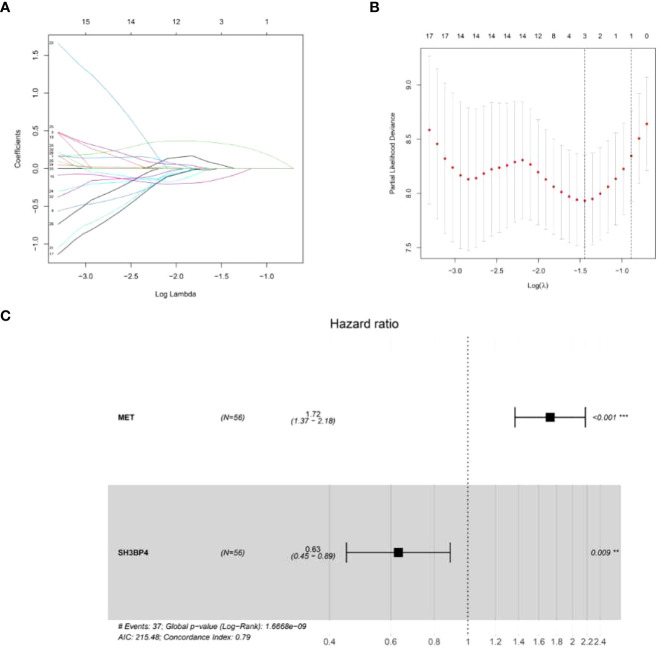
Establishment of a risk score prognostic model. **(A)** LASSO coefficients of the 3 autophagy-associated DEGs. **(B)** Cross-validation for selecting key genes. **(C)** The forest plot of 2 autophagy-associated DEGs in the risk score prognostic model.

**Figure 4:**
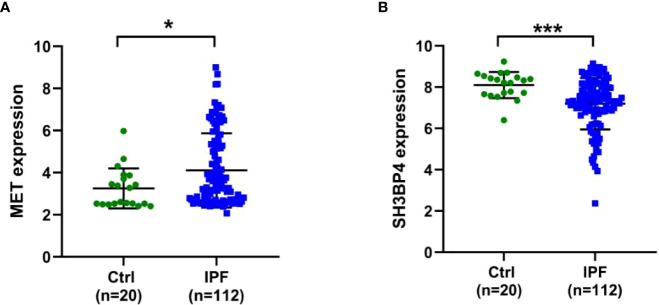
Expression levels of two genes in Ctrl and IPF groups. **(A)** MET. **(B)** SH3BP4. *p<0.05; ***p<0.001.

IPF samples (n=112) were randomly divided into the training set (n=56) and test set (n=56) (**Table 1**). First, 165 autophagy-related DEGs were included for a univariate Cox regression analysis. The results showed that 39 autophagy-related genes were associated with prognosis of patients with IPF (*p*<0.05) (**Supplementary Table 4**). LASSO and multivariate Cox regression analyses were performed to select the key genes from the above 39 genes for construction of a risk score prognostic model. Finally, two genes were identified to establish the risk score prognostic model using the following formula: Risk score= MET × 0.545 + SH3BP4 × (-0.461). In IPF, MET is a risk factor with HR>1, whereas SH3BP4 is a protective factor for HR<1 (**Figure 3** and **Table 2**). In addition, upregulated MET expression and downregulated SH3BP4 expression were found in IPF group as compared with the control group (**Figure 4**).

**Table 2:** Details of the two genes from the risk score prognostic model.

Gene	Location	Coefficient	HR	*P* value
MET	chr7:116,672,196-116,798,386	0.545	1.724	<0.001
SH3BP4	chr2:234,952,017-235,055,714	-0.461	0.631	0.009

HR, hazard ratio.

**Figure 5:**
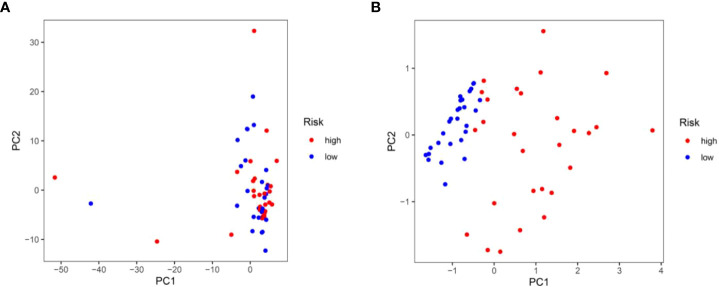
Principal component analysis. **(A)** Principal component analysis based upon all autophagy-associated genes in the training test. **(B)** Principal component analysis based upon two autophagy-associated genes from risk score prognostic model.

**Figure 6:**
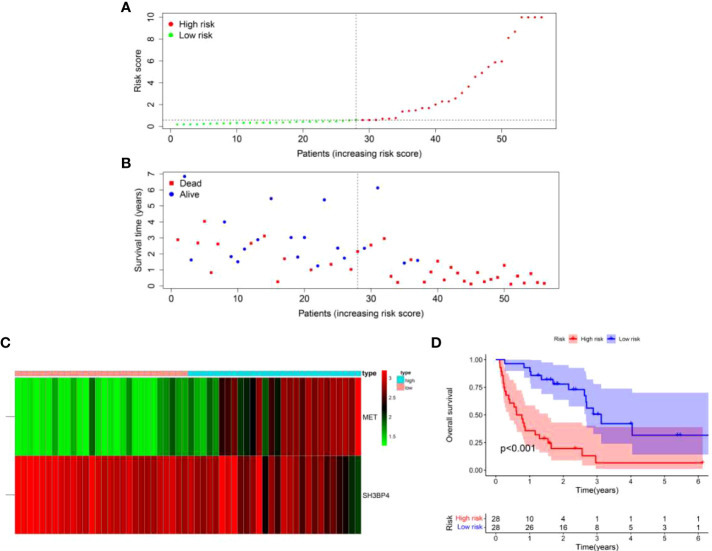
A two-gene risk score model predicted the overall survival in patients with IPF in the training set. **(A)** Distribution of risk score per patient. **(B)** Survival status of each patient. **(C)** Expression heatmap of the two genes. **(D)** Kaplan-Meier survival curve analysis of IPF patients divided into high-risk and low-risk groups.

Based on the formula of risk score, patients were stratified into high-risk and low-risk groups with a median value of risk score as a cut-off point. First, we explored the distribution of patients using PCA analysis. As shown in **Figure 5**, the genes from risk score model could distinguish IPF from different risk groups. In order to assess the performance of risk score model in predicting the prognosis of patients with IPF, Kaplan-Meier curves were conducted. The results demonstrated that patients in high-risk group had a shorter OS as compared to the low-risk group (*p*<0.001) (**Figure 6D**). The distribution of risk score in different risk groups was displayed in **Figure 6A**. Survival status of each patient was shown in **Figure 6B**. The heatmap presented the expression profiles of the two genes in the high-risk and low-risk groups (**Figure 6C**).

### The risk score serves as an independent prognostic indicator

**Figure 7:**
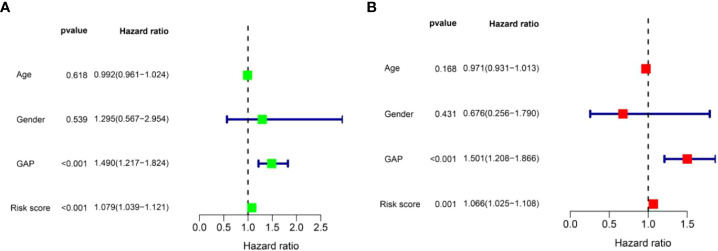
Identification of independent prognostic factors in patients with IPF in the training set. **(A)** The univariate Cox regression analysis for risk score model and clinical parameters. **(B)** The multivariate Cox regression analysis for risk score model and clinical parameters.

In order to determine if the risk score prognostic model is an independent prognostic factor for IPF, univariate and multivariate regression analyses were performed. We integrated the risk score and clinical parameters (including age, sex, and GAP index) for analysis. The univariate regression analysis showed that GAP index and risk score were correlated to prognosis (**Figure 7A**). In the multivariate regression analysis, risk score as well as GAP index was proved to be an independent prognostic indicator (**Figure 7B**). These findings demonstrated that risk score prognostic model is reliable in forecasting the survival of patients with IPF.

**Figure 8:**
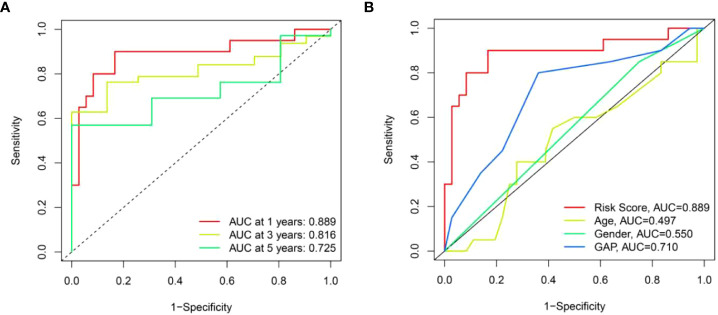
The prognostic value of risk score model in the training set. **(A)** ROC curves of risk score model at 1-, 3-, and 5-year overall survival. **(B)** ROC curves of clinical parameters and risk score model at 1-year overall survival.

We further performed ROC analysis to assess the risk score. The area under the ROC curve (AUC) for risk score at one, three, and five years was 0.889, 0.816, and 0.725, respectively (**Figure 8A**). In addition, we found that the AUC value for risk score at one year (0.889) was higher than age (0.497), sex (0.550) and GAP index (0.710) (**Figure 8B**). Taken together, this risk score model had a good prediction accuracy.

### The relationship between risk scores and clinical features

**Figure 9:**
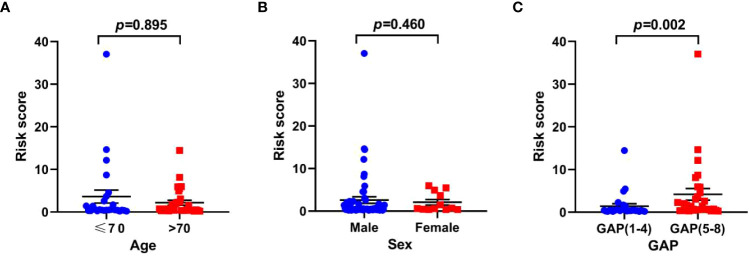
The relationship between risk scores and clinical parameters in the training set. **(A)** age; **(B)** sex; **(C)** GAP index.

To explore the association between risk scores and clinical features, we analyzed the distribution of risk scores in age, sex and GAP index. There was no statistical difference in risk score associations with age and sex, while higher risk scores were related to high GAP index (**Figure 9**).

### Comparison analysis of immune cells or immune functions between high-risk and low-risk groups

**Figure 10:**
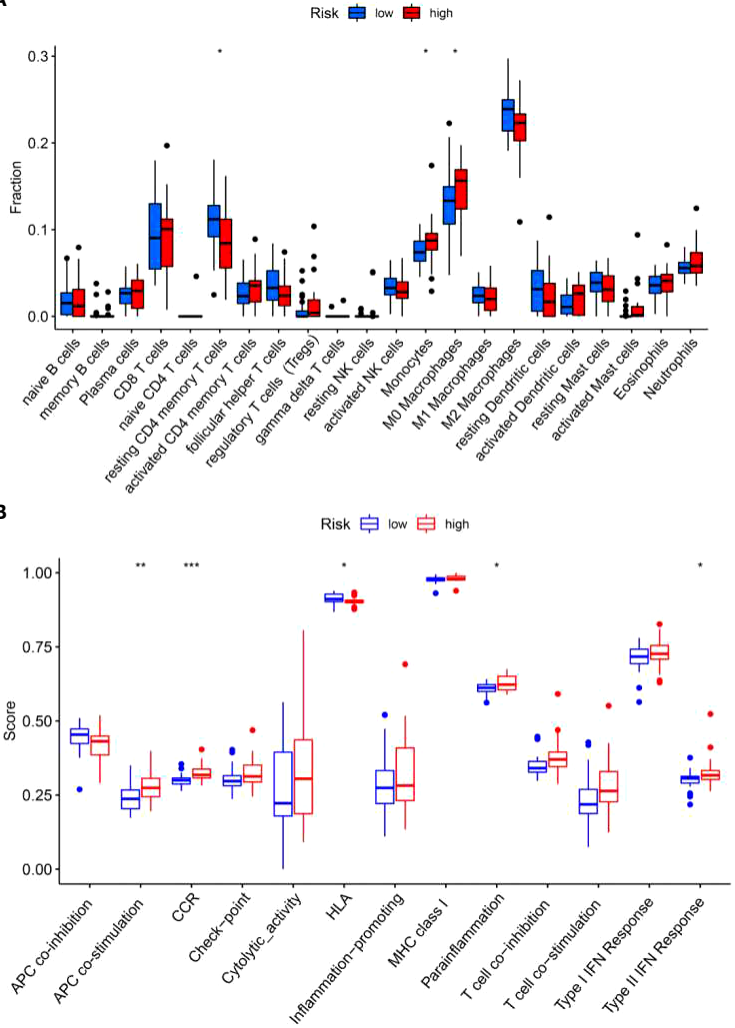
Immune cells and immune-related functions of the two risk groups in the training set. The proportion of 22 types of immune cells **(A)** and 13 immune-related functions **(B)** were analyzed in the high-risk and low-risk group. *p<0.05; **p<0.01; ***p<0.001.

Studies have shown that BALF contains different kinds of blood cells which might affect the progression of pulmonary fibrosis (16). Therefore, we explored the infiltration level of immune cells in high-risk and low-risk groups. We found more monocytes and macrophages in the high-risk group than in the low-risk group (**Figure 10A**). On the contrary, low-risk group has more resting CD4^+^ T cells as compared with high-risk group (**Figure 10A**). In addition, high-risk group exhibits high scores of antigen-presenting cell (APC) co-stimulation, cytokine-cytokine receptor (CCR) interaction, parainflammation and type II IFN response (**Figure 10B**), while,human leukocyte antigen (HLA) was increased in low-risk group (**Figure 10B**).

### GSVA

**Figure 11:**
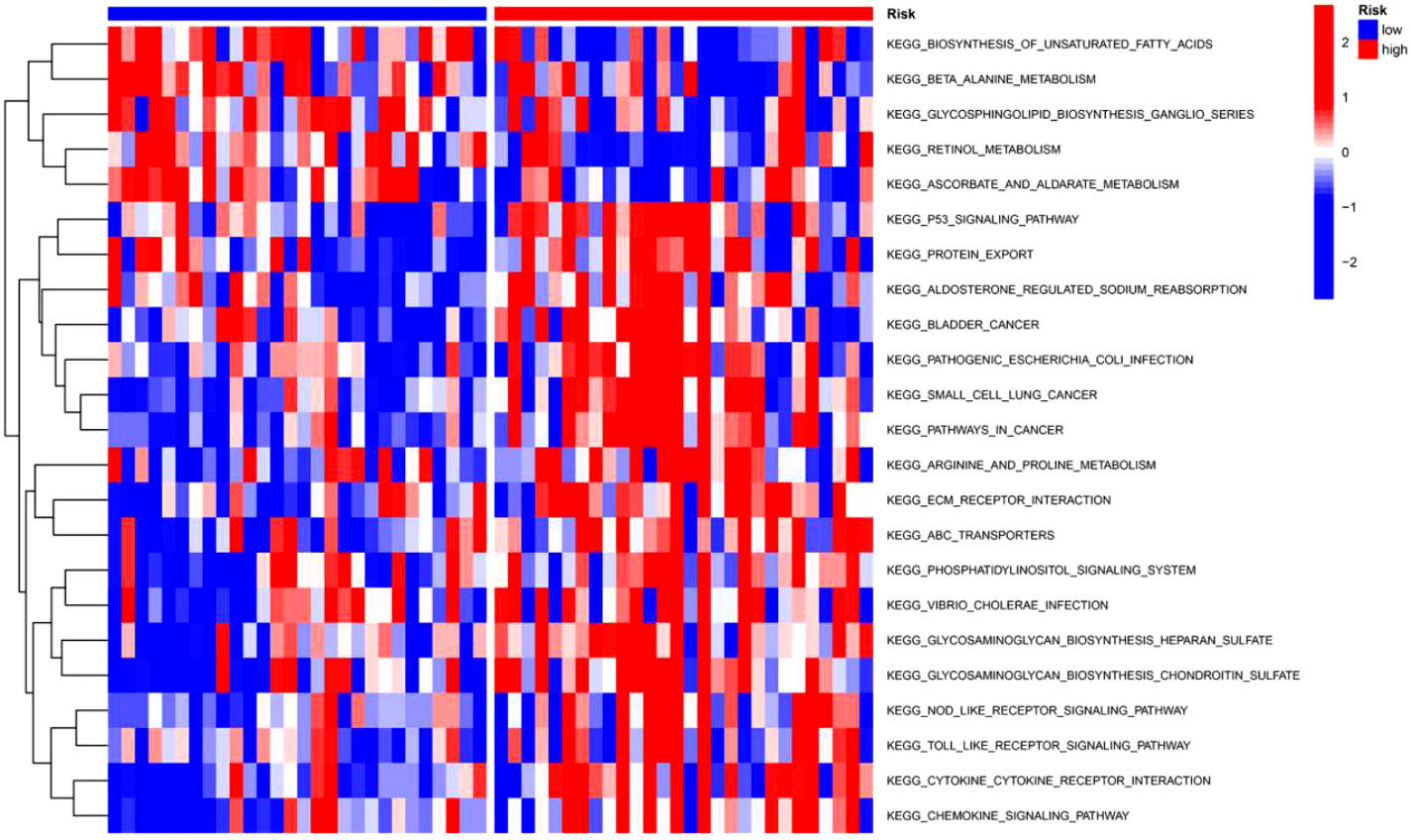
GSVA enrichment analysis between the two risk groups in the training set.

In order to explore the differences in pathway activity between the high-risk and low-risk groups in the training set, GSVA was performed. A total of 23 pathways were found to be statistically significant, such as pathways related to cancer, p53 signaling pathway, cytokine-cytokine receptor interaction, ECM receptor interaction, and Toll-like receptor signaling pathway (**Figure 11**). These pathways were closely linked to the development of IPF (17–21).

### Verification of the risk score model in the test set

**Figure 12:**
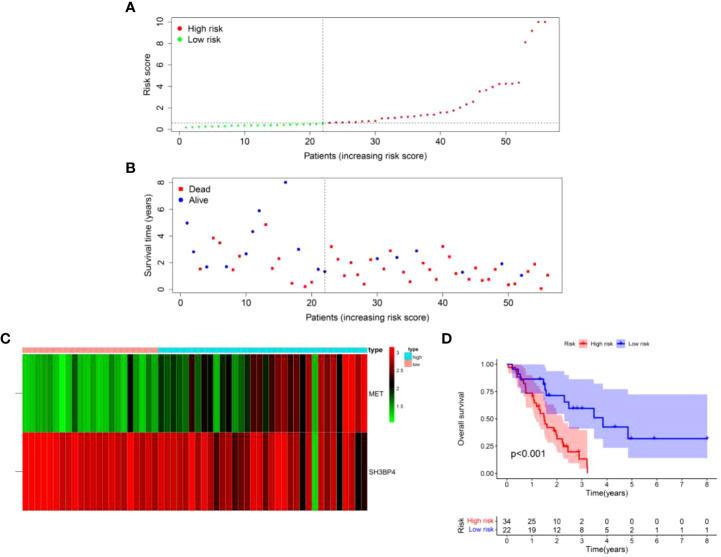
Validation of risk score model in the test set. **(A)** Distribution of risk score per patient. **(B)** Survival status of each patient. **(C)** An expression heatmap of the two genes. **(D)** Kaplan-Meier survival curve analysis of IPF patients divided into the high-risk and low-risk groups.

**Figure 13:**
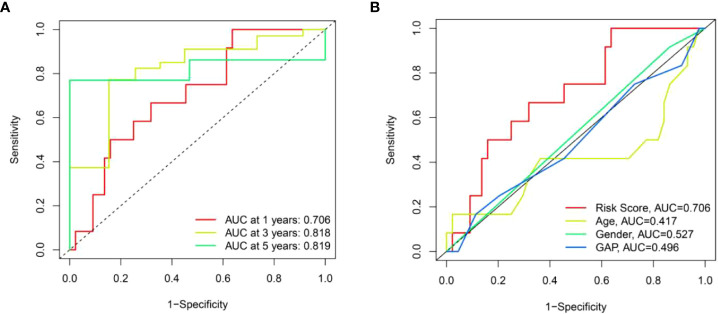
The prognostic value of risk score model in the test set. **(A)** ROC curves of risk score model at 1-, 3-, and 5-year overall survival. **(B)** ROC curves of clinical parameters and risk score model at 1-year overall survival.

To further validate the universality of the risk score model from the training set, the formula was applied in the test set. The risk score of each patient in the test set was calculated according to the formula from the training set. Subsequently, patients in the test set were divided into high-risk and low-risk groups based on the median value of risk score from the training set. The patients’ risk curve and distribution of survival status in the test set were analyzed. We discovered that risk curve, survival status, and heat map were similar to those in the training set (**Figures 12A–C**). Likewise, we found that patients in high-risk group had poorer OS than low-risk group in the test set (**Figure 12D**). In addition, the AUC value was 0.706 in one year, 0.818 in three years and 0.819 in five years, respectively (**Figure 13A**). Furthermore, the AUC value of risk score at one year was better than age, sex, and GAP index (**Figure 13B**). These findings proved the universality and robustness of the risk score prognostic model.

### Validation of model gene expression in clinical specimens and fibroblasts

**Figure 14:**
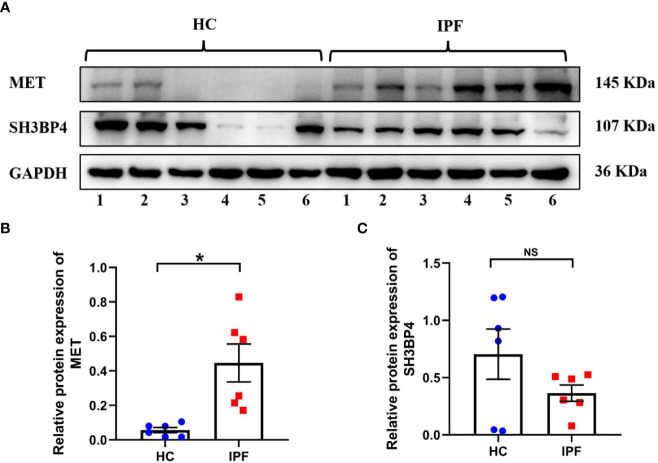
The expression of two model genes in HC and IPF lung tissues. **(A–C)** The protein expression of MET and SH3BP4 in healthy control and IPF lung tissues was detected by western blot assay. HC, healthy control. *p<0.05. NS, not significant. n=6.

To validate the gene expression, we performed qPCR and western blot analysis in clinical specimens and TGF-β1-activated human lung fibroblasts. As shown in **Figures 14A–C**, the protein expression of MET was increased in IPF lung tissues as compared with normal lung tissues. On the contrary, the protein expression of SH3BP4 in IPF lung tissues was decreased as compared to the normal lung tissues, although no statistical significance was observed. This could be due to the relative small sample size or different types of the samples as the microarray data were obtained from BALF cells, while our samples were lung homogenates. The differential expression of autophagy-related genes in the BALF may be coming from different cell types in the BALF between control subjects and IPF patients or from different gene expression profiles within the same types of cells, or combination of these factors, which needs further study.

**Figure 15:**
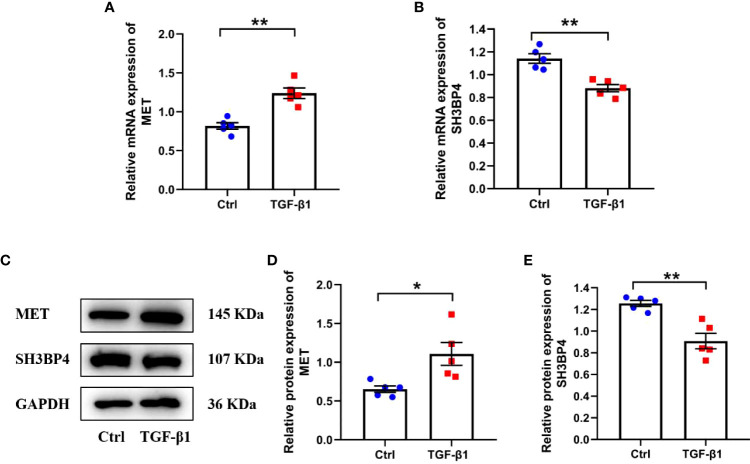
The expression of two model genes in human lung fibroblasts. Human lung fibroblasts were treated with 10ng/ml TGF-β1 for 48h. **(A, B)** The mRNA expression of MET and SH3BP4 was detected by qPCR. **(C–E)** The protein expression of MET and SH3BP4 was detected by western blot assay. *p<0.05; **p<0.01. n=5.

Activated lung fibroblast are the principal effector cells of progressive fibrotic process in IPF (22). TGF-β1, a well-known pro-fibrotic factor, was used to activate fibroblasts. Similar as in IPF lung tissues, we found an upregulated MET expression and downregulated SH3BPE expression in the TGF-β1-activated fibroblasts (**Figures 15A–E**). These results are consistent with the microarray data analysis.

## Discussion

IPF is a progressive lung disease with a poor prognosis. Molecular signatures of gene expression from lung tissue are associated with prognosis of IPF (23). Nevertheless, genomic signatures are not widely applied in clinics as lung biopsy is an invasive and unpleasant operation. Bronchoalveolar lavage (BAL) is a medical procedure that sterile saline solution is injected into lung and then collected by a bronchoscope (24). BAL fluid exhibits biochemical changes due to lung diseases and external factors (24). The analysis of cells in BAL fluid may be conducive to diagnose and treat lung diseases, even assess the prognosis of patients with lung disease (25). Therefore, establishment of a multi-gene prognostic model based on the BALF cells is necessary to predict the prognosis of patients with IPF.

In this study, a total of 165 autophagy-related DEGs was determined between IPF BAL samples and normal BAL samples in the GSE70866 data set. Among the 165 DEGs, 39 DEGs were further identified to be related to prognosis of patients with IPF in the training set. Subsequently, we constructed a two-gene risk score prognostic model based on the LASSO and multivariate Cox regression analyses and further validated it in the test set. Moreover, this risk score model could serve as an independent indicator for patients with IPF. Additionally, the ROC curve indicated that this risk score model had a reliable and effective prediction accuracy. Finally, we found an increased MET expression and decreased SH3BP4 expression in both IPF lung tissues and TGF-β1-activated human lung fibroblasts, consistent with the microarray results. These findings may aid clinicians in identifying high-risk patients and designing individualized treatment strategy for them.

The prognostic model in the present study was composed of two autophagy-related genes (MET and SH3BP4). Receptor tyrosine kinase MET, also known as c-MET, is a receptor of hepatocyte growth factor (HGF) (26). It has been reported that HGF/c-MET signaling pathway participates in multiple cellular processes, including cell survival, proliferation, motility, invasion and metastasis (27). In addition, MET is tightly linked to the process of autophagy (28–30). A number of studies have indicated that MET is closely involved in fibrotic diseases. Marquardt et al. reported that lack of c-MET can promote carbon tetrachloride-induced liver fibrosis in mice (31). Another study has shown an increased MET expression in lung fibroblasts from patients with pulmonary fibrosis as compared with lung fibroblasts from normal people (32). Moreover, MET has been implicated in driving profibrotic phenotypes and leading to pulmonary fibrosis (33, 34). Activation of lung fibroblast plays a major role in the pathogenesis of IPF (22). TGF-β1 has been considered as the main growth factor involved in the differentiation of lung fibroblasts into myofibroblasts (35). In agreement with previous studies, we found that MET expression was upregulated in both IPF lung tissues and TGF-β1-activated human lung fibroblasts, indicating that MET may promote the progression of IPF. SRC homology 3 domain-binding protein 4 (SH3BP4), also known as transferrin receptor trafficking protein (TTP), was first discovered in human corneal fibroblasts (36). SH3BP4 affects autophagy process by negatively regulating Rag GTPase- mTOR complex 1 (mTORC1) signaling pathway (37). Besides, SH3BP4 negatively regulates Wnt signaling *via* modulating β-catenin’s subcellular localization, thus suppressing tumor development (38). Kim et al. reported that SH3BP4 is a direct target gene of miR-125b and is negatively regulated by miR-125b (39). In another study, upregulated miR-125b was found in both human cardiac fibrosis and TGF-β-treated human cardiac fibroblasts (40). Consistent with the microarray data, we observed decreased expression of SH3BP4 in IPF lung tissues and TGF-β1-activated human lung fibroblasts. We speculated that SH3BP4 is a negative regulator in the occurrence and progression of IPF and the underlying mechanism needs to be further elucidated.

Increasing evidences indicate that immune cells are linked to the development of IPF (1, 19, 41, 42). Kreuter et al. reported that increased monocyte count was related to elevated risks of IPF progression, hospitalization and mortality for patients with IPF (43). Another study also indicated that high absolute monocyte count is an IPF specific marker of mortality and poor outcomes (44). Macrophages play important roles in IPF. Single-cell transcriptomic analysis identified a distinct population of profibrotic alveolar macrophages exclusively in patients with pulmonary fibrosis (45). Also, accumulation of CD163^+^ and CD204^+^ macrophages in lung leads to worse clinical course in IPF patients (46). Consistent with these studies, we found a higher amount of monocytes and macrophages in high-risk group than low-risk group, indicating that the elevated monocytes and macrophages may be related to the progression of IPF. We also found a reduced amount of resting memory CD4^+^ T cells in BALF of the high-risk group as compared with the low-risk group, indicating that resting memory CD4^+^ T cells may exhibit a protective role in IPF. Besides, significant differences in APC co-stimulation, CCR, HLA, parainflammation and type II IFN response were identified between the high-risk and low-risk groups. The exact role of these immune cells in the pathogenesis of IPF awaits further study.

There are limitations in the present study. First of all, the construction and validation of prognostic model were based on the retrospective data from GEO database, and the sample size in cohort was relatively small. Thus, a prospective study of large sample size is necessary to identify its clinical application. Second, clinical information was not complete in the data set, such as patients’ therapy approaches, laboratory test, lung function data and so on, therefore, the significance of prognostic model was restricted. Last but not least, the association between risk score and immune activity needs to be further explored in basic experiments.

## Conclusion

In summary, our study identified a novel risk score prognostic model of two autophagy-related genes, providing a new approach to predict the prognosis of IPF patients.

## Data availability statement

The original contributions presented in the study are included in the article/**Supplementary Material**. Further inquiries can be directed to the corresponding authors.

## Ethics statement

This study was approved by the ethics committee of the First Affiliated Hospital of Guangzhou Medical University and was carried out in accordance with the Declaration of Helsinki. All subjects have provided written informed consent. IPF patients included in this study were according to the diagnostic criteria of 2018 ATS/ERS/JRS/ALAT Clinical Practice Guideline (47).

## Author contributions

XT, NZ and GH conceived the study. XT designed and supervised the study, revised and edited the manuscript. GH performed data analysis and drafted the manuscript. XX, CJ and JH contributed to clinical specimen collection for validation. All authors reviewed and approved the final version of the manuscript.

## Funding

This study was supported by the National Natural Science Foundation of China (XT), the National High-Level Talents Program (XT), Local Innovative and Research Teams Project of Guangdong Pearl River Talents Program (2017BT01S155), Open Project of State Key Laboratory of Respiratory Disease (SKLRD-OP-202109), Guangzhou Institute of Respiratory Health Open Project (XT) and Postdoctoral Startup Fund of Guangzhou City (GH).

## Acknowledgments

We would like to express our gratitude to the GEO databases for providing us with this excellent public data.

## Conflict of interest

The authors declare that the research was conducted in the absence of any commercial or financial relationships that could be construed as a potential conflict of interest.

## Publisher’s note

All claims expressed in this article are solely those of the authors and do not necessarily represent those of their affiliated organizations, or those of the publisher, the editors and the reviewers. Any product that may be evaluated in this article, or claim that may be made by its manufacturer, is not guaranteed or endorsed by the publisher.
