# Supplementary material for: Identification and validation of autophagy-related gene expression for predicting prognosis in patients with idiopathic pulmonary fibrosis
Source: Front Immunol. 2022 Sep 20;13:997138. doi: 10.3389/fimmu.2022.997138 (PMC9533718; doi:10.3389/fimmu.2022.997138)
Supplement: Supplementary file 1 [file DataSheet_1.pdf]

Supplementary Table 1. A total of 12 autophagy-related gene sets downloaded from the Molecular Signatures Database

Autophagy-related gene set	Organism	Genes (n)
1) GOBP_CHAPERONE_MEDIATED_AUTOPHAGY.gmt	Homo sapiens	16
2) GOBP_NEGATIVE_REGULATION_OF_AUTOPHAGY.gmt	Homo sapiens	87
3) GOBP_POSITIVE_REGULATION_OF_AUTOPHAGY.gmt	Homo sapiens	136
4) GOBP_REGULATION_OF_AUTOPHAGY.gmt	Homo sapiens	339
5)GOBP_REGULATION_OF_AUTOPHAGY_OF_MITOCHONDRION_IN _RESPONSE_TO_MITOCHONDRIAL_DEPOLARIZATION.v7.5.1.gmt	Homo sapiens	14
6) GOBP_SELECTIVE_AUTOPHAGY.gmt	Homo sapiens	77
7) KEGG_REGULATION_OF_AUTOPHAGY.gmt	Homo sapiens	35
8) REACTOME_AUTOPHAGY.gmt	Homo sapiens	151
9) REACTOME_CHAPERONE_MEDIATED_AUTOPHAGY.gmt	Homo sapiens	22
10) REACTOME_SELECTIVE_AUTOPHAGY.gmt	Homo sapiens	82
11) WP_AUTOPHAGY.gmt	Homo sapiens	30
12) WP_HOSTPATHOGEN_INTERACTION_OF_HUMAN_ CORONAVIRUSES_AUTOPHAGY.gmt	Homo sapiens	19
Removing overlapping genes		504

Supplementary Table 2. A total of 504 autophagy-related genes from the Molecular Signatures Database

ATG5	LRRK2	ADRB2	NPRL2	TP53INP2
ATP13A2	LZTS1	AMBRA1	NPRL3	TPCN1
BAG3	MAGEA3	ATF6	OPTN	TRIM13
CLU	MAGEA6	ATG16L1	ORMDL3	TRIM14
CTSA	MCL1	ATG2A	PAFAH1B2	TRIM21
EEF1A1	MET	BAD	PARK7	TRIM22
EEF1A2	MIR199A1	BCL2L11	PIK3C2A	TRIM27
GFAP	MIRLET7B	BNIP3	PIK3CB	TRIM32
HSP90AA1	MT3	BNIP3L	PIM2	TRIM34
HSPA8	MTM1	C9orf72	PIP4K2A	TRIM38
LAMP2	MTMR8	CALCOCO2	PIP4K2B	TRIM5
PLK3	MTMR9	CAMKK2	PIP4K2C	TRIM6
SNCA	MTOR	CERS1	PLEKHF1	TRIM65
SNRNP70	NPC1	DAPK1	PLK2	TRIM68
STUB1	NRBP2	DCN	PRKAA1	TRIM8
SYNPO2	NUPR1	DEPDC5	PRKAA2	TRIML1
ADRA1A	PHF23	DHRSX	PRKD1	TRIML2
AKT1	PIK3CA	ELAPOR1	PRKN	UFL1
ATG7	PINK1	ENDOG	RAB3GAP1	ULK1

BCL2	POLDIP2	EPM2A	RAB3GAP2	ULK2
BECN1	PTPN22	FBXO7	RALB	UVRAG
BMF	QSOX1	FLCN	RIPK2	VDAC1
CHMP4A	RASIP1	FOXO1	RNF152	VPS13D
CHMP4B	RNF41	FOXO3	RNF31	WAC
CLEC16A	RNF5	FYCO1	ROCK1	WDR45
CPTP	RRAGA	GNAI3	RUFY4	WIPI1
DAP	RUBCN	GPSM1	SCOC	ZC3H12A
DAPL1	SCFD1	GSK3A	SESN1	ABL1
EHMT2	SEC22B	GSK3B	SESN2	ABL2
EIF4E	SIRT2	HIF1A	SESN3	ACER2
EIF4G1	SMCR8	HMGB1	SH3BP4	ATG12
EIF4G2	SMG1	HSPB8	SH3GLB1	ATG14
EIF4G3	STAT3	HTT	SIRT1	ATM
FEZ1	TAB2	IFNG	SLC25A4	ATP6V0A1
FEZ2	TAB3	IKBKG	SLC25A5	ATP6V0A2
FOXK1	TBC1D14	IL4	SNX18	ATP6V0B
FOXK2	TIGAR	IRGM	SNX30	ATP6V0C
GATA4	TLK2	KAT5	SNX4	ATP6V0D1
GOLGA2	TMEM39A	KDR	SNX7	ATP6V0D2
HERC1	TP53	LACRT	SPTLC1	ATP6V0E1
HGF	TREM2	LARP1	SPTLC2	ATP6V0E2
HMOX1	TSC1	LRSAM1	STING1	ATP6V1A
HTR2B	TSC2	MAP3K7	STK11	ATP6V1B1
IL10	TSPO	MAPK3	SUPT5H	ATP6V1B2
IL10RA	UBQLN4	MEFV	SVIP	ATP6V1C1
KDM4A	USP30	MID2	TBK1	ATP6V1C2
KIF25	USP36	MOAP1	TFEB	ATP6V1D
KLHL22	WASHC1	MTDH	TICAM1	ATP6V1E1
LEP	WDR6	NOD1	TMEM59	ATP6V1E2
LEPR	ZKSCAN3	NOD2	TP53INP1	ATP6V1G1
ATP6V1G2	MTMR3	MUL1	IFNA6	PLIN3
ATP6V1H	MTMR4	TOMM7	IFNA7	PRKAB1
BOK	NEDD4	ACBD5	IFNA8	PRKAB2
CAPN1	NLRP6	ARFIP2	INS	PRKAG1
CAPNS1	OSBPL7	ATG13	ULK3	PRKAG2
CASP3	PARL	ATG2B	ARL13B	PRKAG3
CDK5	PIK3C3	ATG4B	ATG10	RNASE1
CDK5R1	PIK3R2	ATG4D	ATG101	RPS27A
CISD1	PIK3R4	AUP1	ATG9A	SLC38A9
CISD2	PRKACA	C5orf51	ATG9B	SRC
CLN3	PSAP	DDRGK1	CETN1	TOMM20
CRYBA1	PYCARD	KLHL3	CFTR	TOMM22

CSNK2A2	RAB39B	LGALS8	CHMP2A	TOMM40
CTTN	RAB8A	LRBA	CHMP2B	TOMM5
DAPK2	RHEB	MAP1LC3B	CHMP3	TOMM6
DAPK3	RMC1	MAP1LC3C	CHMP4C	TOMM70
DCAF12	RPTOR	PHB2	CHMP6	TSG101
DDIT3	RRAGB	PJVK	CHMP7	TUBA1A
DEPP1	RRAGC	RAB7A	CSNK2A1	TUBA1B
DNM1L	RRAGD	RB1CC1	CSNK2B	TUBA1C
DRAM1	SNX32	RETREG1	DYNC1H1	TUBA3C
DRAM2	SNX5	RETREG3	DYNC1I1	TUBA3D
EP300	SNX6	RNF213	DYNC1I2	TUBA3E
ERCC4	SOGA1	SQSTM1	DYNC1LI1	TUBA4A
ERN1	SOGA3	STBD1	DYNC1LI2	TUBA4B
EXOC1	SREBF1	TAFAZZIN	DYNLL1	TUBA8
EXOC4	SREBF2	TEX264	DYNLL2	TUBAL3
EXOC7	TBC1D25	UBA5	EPAS1	TUBB1
EXOC8	TECPR1	UFC1	FUNDC1	TUBB2A
FBXL2	TPCN2	UFM1	HBB	TUBB2B
FBXW7	TRIB3	WDFY3	HSF1	TUBB3
FZD5	UBQLN1	WDR81	HSP90AB1	TUBB4A
GAPDH	UBQLN2	WIPI2	IFT88	TUBB4B
GBA	UCHL1	ATG3	LAMTOR1	TUBB6
GPR137	USP10	ATG4A	LAMTOR2	TUBB8
GPR137B	USP13	ATG4C	LAMTOR3	TUBB8B
HAX1	USP33	BECN2	LAMTOR4	UBA52
HDAC6	VPS13C	GABARAP	LAMTOR5	UBAP1
HSPB1	VPS26A	GABARAPL1	MAP1LC3A	UBB
HTRA2	VPS26B	GABARAPL2	MFN1	UBC
IFI16	VPS29	IFNA1	MLST8	UBE2N
ITPR1	VPS35	IFNA10	MTERF3	UBE2V1
KAT8	WDR24	IFNA13	MTMR14	VCP
KEAP1	WDR41	IFNA14	MVB12A	VIM
LAMP3	ZMPSTE24	IFNA16	MVB12B	VPS28
MAPK15	ATP5IF1	IFNA17	NBR1	VPS37A
MAPK8	CDC37	IFNA2	PCNT	VPS37B
MAPT	HK2	IFNA21	PEX5	VPS37C
MFSD8	HUWE1	IFNA4	PGAM5	VPS37D
MTCL1	MFN2	IFNA5	PLIN2	WDR45B
AKT1S1	DEPTOR	ATG16L2	ZFYVE1	

Supplementary Table 3. The autophagy-related differentially expressed genes between Ctrl and IPF group (n=165)

Gene	Ctrl-Mean	IPF-Mean	log FC	*p* Value
HSP90AA1	15.43368555	15.16485376	-0.025351032	0.022692443
LAMP2	13.2611766	13.03392063	-0.02493767	0.026761305
PLK3	11.4525815	11.77542777	0.04010664	0.016581636
SNCA	7.65568405	8.74098842	0.191265136	1.43E-06
ADRA1A	8.62046825	8.921814179	0.049570864	0.002017591
ATG7	9.81966205	10.06615597	0.035767578	0.000786888
BECN1	11.2358168	11.04518526	-0.02468739	0.033229733
CLEC16A	9.42012505	9.160024098	-0.040394818	0.00805608
FEZ1	2.64221065	2.97280042	0.170077121	0.000454029
FEZ2	11.26351375	10.88664183	-0.04909796	0.001608978
FOXK1	6.9299914	6.723097089	-0.043727578	0.026329303
GOLGA2	7.9121424	7.617462438	-0.054757911	0.025692127
HERC1	10.33823475	10.00448878	-0.047342419	0.000397982
HGF	3.19063345	3.659638241	0.197858167	0.013317078
IL10	5.0436665	5.639715455	0.161149491	0.010633803
IL10RA	13.5225867	13.14467014	-0.040893208	0.000709179
KIF25	2.8864474	2.597698491	-0.152060945	0.00868203
LEP	2.91143495	3.562841098	0.291297756	0.009011153
LEPR	14.8250411	14.56287549	-0.025740856	0.039443055
LRRK2	6.51364055	5.905220911	-0.141473077	0.008927599
MET	3.2544508	4.112077661	0.337453407	0.039449225
MT3	10.7618705	11.14712771	0.050743165	0.001812346
MTM1	6.1290279	5.648275446	-0.117847827	0.004465206
MTMR8	5.42319905	5.108489768	-0.086247276	0.005179026
MTMR9	7.4265323	7.050124509	-0.075039988	0.042586543
NRBP2	6.0635154	5.572553545	-0.121815883	0.04768719
NUPR1	15.60069075	15.03755849	-0.05303956	0.000464659
POLDIP2	10.9734796	11.05688231	0.010923584	0.01573933
PTPN22	9.66109235	9.248574071	-0.062955369	0.000892289
QSOX1	14.8893907	14.47126656	-0.041093522	0.000653798
SCFD1	11.1032457	10.80785175	-0.038901676	0.003726624
STAT3	8.7754662	8.51305783	-0.04379834	0.003470888
TMEM39A	7.72930565	8.063518616	0.061070696	0.003689288
TREM2	11.75628395	12.46412315	0.084349283	7.89E-06
TSPO	17.2803611	16.9771787	-0.025536636	0.018966574
ADRB2	10.75090975	10.46929923	-0.038293869	0.003037836
ATF6	9.9698105	10.20264896	0.033305787	0.000476172
ATG2A	11.05746235	10.72554364	-0.043969555	0.005766102
CALCOCO2	8.2611277	7.822181688	-0.078767687	5.07E-05
DCN	2.54615475	3.078338438	0.273831747	0.011855214

DEPDC5	7.4359928	7.082185268	-0.070330789	0.00635191
FLCN	8.57342395	8.809149964	0.039131328	0.002467181
FOXO1	8.8783973	8.531404027	-0.057516082	0.001118149
FYCO1	9.38811495	9.137762536	-0.038994555	0.002173664
GPSM1	10.5073203	10.94156949	0.058424914	0.00115624
HIF1A	8.42984825	8.761757688	0.055713656	0.020017104
HSPB8	2.8438451	3.835226545	0.431468914	0.037082082
HTT	9.45647965	9.072506563	-0.059802018	0.000348477
IFNG	6.7670182	5.39131125	-0.327884067	0.005179026
LACRT	2.75525165	3.293946768	0.257633148	0.010065982
LARP1	12.4740785	12.19439178	-0.032715441	0.000304663
LRSAM1	8.03494015	7.780798134	-0.046369127	0.009437896
MAP3K7	9.28453325	9.189398295	-0.014858986	0.04006034
MID2	6.2893834	5.441863723	-0.208817756	0.007192812
NOD1	8.41938965	8.134462268	-0.049668672	0.013198732
NOD2	10.4763559	9.93041	-0.077211787	1.59E-05
OPTN	9.1925517	8.666320536	-0.085045788	0.000833297
ORMDL3	11.64824145	11.86950954	0.027148157	0.008927778
PIM2	8.75262505	8.414738313	-0.056797362	0.006792906
PIP4K2A	11.17023275	10.91425511	-0.033445577	0.002796487
PIP4K2B	10.89903685	10.63168619	-0.035830223	0.000575173
PRKAA1	7.7856108	7.160494366	-0.120751031	3.86E-05
RAB3GAP1	10.513738	10.16386864	-0.048826054	0.000151296
RNF31	9.9725959	9.7672235	-0.030020582	0.017765992
ROCK1	10.09585995	9.800765661	-0.042797437	0.031687926
SESN2	8.67656305	8.342505902	-0.056642875	0.012399652
SH3BP4	8.1031123	7.200408589	-0.17039736	0.000454052
SNX4	8.6398418	8.335765036	-0.051690284	0.008363945
SNX7	9.4591581	8.844466545	-0.096936655	0.001975007
SPTLC2	7.0421055	6.473814866	-0.121390732	0.001773811
TFEB	11.09317755	11.44269779	0.044754557	0.011855104
TICAM1	12.1337008	11.73355021	-0.048380047	0.000173997
TMEM59	13.57160125	13.37331662	-0.021233646	0.013793951
TP53INP1	7.8251661	8.420812491	0.105838064	0.014547381
TPCN1	7.4889613	8.095368777	0.112331168	0.030207245
TRIM13	9.9831341	9.632727955	-0.051548383	7.59E-05
TRIM14	11.4836119	11.06575454	-0.053474652	3.09E-05
TRIM22	11.3110233	10.87279971	-0.057005977	6.03E-06
TRIM32	6.3635674	6.026112643	-0.078608123	0.009882768
TRIM38	8.72044535	8.233492679	-0.082897258	1.11E-05
TRIM6	6.47792895	5.827576295	-0.152636657	0.001130687
TRIM65	10.94256935	10.49768157	-0.059880786	1.32E-05
TRIM8	11.068416	10.98957032	-0.010313793	0.01643849

TRIML1	2.1050058	2.70839717	0.363615108	0.000374628
TRIML2	2.73099095	2.664130473	-0.035759795	0.014044019
ULK2	8.1573344	8.388504518	0.040315839	0.018228551
UVRAG	7.17585525	6.634980071	-0.113058656	8.89E-05
VDAC1	12.95885335	13.10160639	0.015805643	0.002219833
VPS13D	5.8747185	5.51129767	-0.092127673	0.000805184
WIPI1	10.68560385	10.93966837	0.033900566	0.014168396
ABL2	8.11086505	8.448261893	0.058798768	0.001277844
ATP6V0B	13.9018066	14.08973201	0.019371792	0.011536487
ATP6V0D2	5.75649065	6.52295242	0.180335541	0.004163675
ATP6V0E2	7.37685465	7.146364366	-0.045796336	0.041623226
ATP6V1B1	3.0965595	2.690037366	-0.203039955	0.011228551
ATP6V1E2	7.32668255	6.918001214	-0.082804841	0.001277844
ATP6V1G1	12.90967005	12.73991973	-0.01909594	0.012849012
BOK	7.2639535	7.633630884	0.071614461	0.014676458
CDK5	10.20918425	10.37368488	0.023060856	0.003506551
DNM1L	8.7252511	8.417066536	-0.051879129	0.018861888
DRAM1	11.9217083	11.56679194	-0.043602192	0.001365348
EP300	8.07340595	7.626868723	-0.082086568	0.001263749
EXOC7	11.2942278	11.13468749	-0.020524568	0.020014839
EXOC8	8.33618665	8.002126393	-0.059004164	0.004205347
FBXL2	5.98004425	5.463638455	-0.13029414	0.007905944
FBXW7	8.56149665	8.279399455	-0.048336893	0.021232748
ITPR1	9.8077924	9.316621607	-0.074121542	0.000631652
MAPT	3.6300984	3.220869991	-0.172558227	0.011331762
NEDD4	4.8504417	5.287432482	0.124451205	0.001105673
NLRP6	3.1104917	3.121294116	0.005001651	0.024057013
PSAP	16.06148105	15.71891159	-0.031103606	0.003724465
RAB39B	4.90833795	3.757095196	-0.385616914	1.28E-06
RAB8A	12.2594994	12.08571663	-0.020597048	0.022511328
RPTOR	9.814273	9.537215884	-0.041313229	0.01082924
RRAGB	6.5801456	6.006491375	-0.131597004	0.004833676
SREBF1	12.54774855	12.15235638	-0.04619244	0.001933065
SREBF2	9.4667789	9.620582527	0.023250624	0.027870337
TECPR1	10.5649082	10.25887577	-0.042407592	0.001933065
TRIB3	10.6926578	11.22553542	0.07016376	0.00340101
UCHL1	6.30846505	7.335092902	0.217526221	0.000118497
VPS13C	10.4694745	9.854271384	-0.087367921	0.000143753
HK2	12.5103274	12.05164742	-0.053889174	1.57E-05
HUWE1	9.9042152	9.717516589	-0.027454996	0.039447425
TOMM7	15.62031665	15.39152714	-0.021287317	0.010623728
ACBD5	9.86985025	9.460727196	-0.061077115	0.01902351
ATG2B	7.9436962	7.509239839	-0.081143578	0.008763178

LRBA	7.5011336	7.177553607	-0.063616437	0.026762099
UBA5	8.14483135	7.817070955	-0.059256693	0.011961952
WDR81	12.53997535	12.28388014	-0.029768171	0.003435429
ATG3	11.4142398	11.2192833	-0.024854259	0.020878096
GABARAPL1	13.11238365	12.66573976	-0.049998629	1.16E-05
IFNA10	2.30454405	2.553611295	0.148057594	0.003999662
IFNA16	2.6624118	2.533098839	-0.071830362	0.018385571
IFNA7	2.96294635	2.71167692	-0.127847204	0.020186844
IFNA8	2.92411015	2.735765848	-0.096052902	0.011854993
INS	4.2053022	4.584011339	0.124401134	0.022512735
ARL13B	5.37922815	4.723799205	-0.187451538	0.001182272
ATG9B	3.10668115	3.658638813	0.235932817	0.018385571
CFTR	3.025035	2.919713518	-0.051125016	0.041307484
CHMP4C	5.514334	4.539955	-0.280508656	0.000131395
CHMP6	8.50284605	8.837556982	0.055701795	0.000357017
DYNC1I1	2.771233	3.14449675	0.18230113	0.006537613
DYNC1LI2	8.3703982	8.095762304	-0.048129325	0.016156006
EPAS1	13.46783735	13.03190082	-0.047470674	0.000116945
HBB	12.5965485	14.24159746	0.177082497	0.007054295
HSP90AB1	12.21437455	12.08488938	-0.015375724	0.043897566
IFT88	7.5823118	7.139221438	-0.086871033	0.008208596
MAP1LC3A	6.60000945	6.862899277	0.056350091	0.015199977
NBR1	12.35308805	12.18056071	-0.020291189	0.049865218
PCNT	9.5427845	9.229126366	-0.048216205	0.003101236
PEX5	4.96422605	4.316390786	-0.201743326	0.000561859
PRKAB2	7.8122246	7.443925259	-0.069669857	0.019185556
PRKAG3	9.7179791	9.989780589	0.039796662	0.026545235
RNASE1	11.5534096	13.23203162	0.195715909	2.13E-06
SRC	9.2055642	8.701103196	-0.081307817	0.002767685
TOMM40	8.1176248	8.464375973	0.060346051	0.038249962
TOMM6	12.8070853	12.98828037	0.020268255	0.002626714
TSG101	11.80245275	11.64255436	-0.019679089	0.022139881
TUBA3D	7.88346475	8.379893304	0.088102047	0.011961952
TUBB2B	2.71970875	2.617589804	-0.05521313	0.001717121
TUBB3	3.46439105	5.927682259	0.774866331	4.45E-07
UBE2N	8.11416025	7.758872554	-0.064594766	0.00215071
VPS37C	13.0734049	12.54883788	-0.059081167	6.22E-06
AKT1S1	7.9259858	8.050551259	0.022497192	0.007470612
ATG16L2	12.8958315	12.48016099	-0.047268254	0.000272839

Supplementary Table 4. The autophagy-related DEGs associated with prognosis in training test using univariate Cox regression analysis (n=39)

Gene	HR	HR(95% low limit)	HR(95% high limit)	p value
MET	1.874650946	1.505210616	2.334767064	2.00E-08
SH3BP4	0.458239203	0.324639708	0.64681911	9.10E-06
PLK3	4.41677844	2.086518785	9.349511698	0.000103495
GABARAPL1	0.258020305	0.121590411	0.547530658	0.000417035
TRIM14	0.244918841	0.111699159	0.537024979	0.000444749
UCHL1	1.519040577	1.197318586	1.927209937	0.000575255
HK2	0.297825942	0.149119263	0.594827861	0.000599567
TPCN1	1.563991552	1.207345696	2.025989393	0.000706974
TUBB3	1.430729067	1.155746369	1.771137438	0.00100492
IL10	1.631491296	1.209326294	2.201030327	0.001355098
SNCA	1.863928827	1.258399692	2.760832425	0.001892055
RRAGB	0.578598574	0.407765607	0.821001832	0.002178919
TRIB3	2.175228407	1.320039364	3.584452668	0.002292016
EXOC7	0.226150709	0.086080002	0.594146632	0.002558157
SESN2	0.446980986	0.262167984	0.762076278	0.00309536
LEP	1.319604149	1.082842354	1.608133543	0.005980833
LAMP2	0.316465113	0.134768658	0.743126553	0.008251238
HBB	1.211376237	1.050534973	1.396842966	0.0083338
CDK5	0.27227164	0.102799786	0.721128405	0.008848711
HIF1A	2.112826702	1.199736147	3.720848692	0.009579897
NLRP6	1.510378455	1.093754877	2.085698656	0.012273225
SNX7	0.573996307	0.368606988	0.893829393	0.014023147
SRC	0.624857459	0.428944049	0.910251219	0.014290429
QSOX1	0.44772768	0.23391341	0.856984112	0.015271047
ATG7	0.447966716	0.229489136	0.874438685	0.018616113
ADRB2	0.449070966	0.225625142	0.893804348	0.022628439
PSAP	0.62140449	0.411947392	0.937361292	0.023306426
SPTLC2	0.658591942	0.459060467	0.944850138	0.023327575
WIPI1	2.176468104	1.10990179	4.267957264	0.02360876
NUPR1	0.560572958	0.337703243	0.930527166	0.025192905
RNASE1	1.381038948	1.031212316	1.849540146	0.030296435
TRIM38	0.463569267	0.230841598	0.930926086	0.030682263
ABL2	2.305694059	1.066146398	4.986393148	0.033777518
TOMM7	0.49282893	0.256024083	0.948662139	0.034201093
FLCN	2.179103201	1.056816521	4.493202622	0.034889807
NBR1	0.412882608	0.179651569	0.948903753	0.037206083
RAB8A	0.431844184	0.194012559	0.961223334	0.039701731
UBE2N	0.638737019	0.415322418	0.982333151	0.041238139
MTM1	0.664334859	0.443313544	0.995550012	0.047526986

Supplementary Table 5. Significant pathways between the high-risk and low-risk groups (n=23)

id	logFC	*p* value
KEGG_SMALL_CELL_LUNG_CANCER	0.173063	1.5E-05
KEGG_GLYCOSAMINOGLYCAN_BIOSYNTHESIS_HEPARAN_SULFATE	0.189338	3.13E-05
KEGG_GLYCOSAMINOGLYCAN_BIOSYNTHESIS_CHONDROITIN_SULFATE	0.227099	0.000201
KEGG_PATHWAYS_IN_CANCER	0.106039	0.00027
KEGG_BIOSYNTHESIS_OF_UNSATURATED_FATTY_ACIDS	-0.221714	0.000301
KEGG_P53_SIGNALING_PATHWAY	0.173347	0.00083
KEGG_BLADDER_CANCER	0.198861	0.000917
KEGG_CYTOKINE_CYTOKINE_RECEPTOR_INTERACTION	0.157987	0.001079
KEGG_PATHOGENIC_ESCHERICHIA_COLI_INFECTION	0.173713	0.001299
KEGG_ALDOSTERONE_REGULATED_SODIUM_REABSORPTION	0.136003	0.001633
KEGG_ECM_RECEPTOR_INTERACTION	0.13216	0.00221
KEGG_CHEMOKINE_SIGNALING_PATHWAY	0.128149	0.003441
KEGG_ABC_TRANSPORTERS	0.130692	0.003947
KEGG_ARGININE_AND_PROLINE_METABOLISM	0.115428	0.010804
KEGG_RETINOL_METABOLISM	-0.11021	0.011637
KEGG_PROTEIN_EXPORT	0.183259	0.012895
KEGG_NOD_LIKE_RECEPTOR_SIGNALING_PATHWAY	0.137778	0.013396
KEGG_GLYCOSPHINGOLIPID_BIOSYNTHESIS_GANGLIO_SERIES	-0.162673	0.015055
KEGG_PHOSPHATIDYLINOSITOL_SIGNALING_SYSTEM	0.107269	0.015263
KEGG_ASCORBATE_AND_ALDARATE_METABOLISM	-0.164135	0.018358
KEGG_TOLL_LIKE_RECEPTOR_SIGNALING_PATHWAY	0.114389	0.025366
KEGG_BETA_ALANINE_METABOLISM	-0.12658	0.029689
KEGG_VIBRIO_CHOLERAE_INFECTION	0.117771	0.033904
